# Data Centric Storage Technologies: Analysis and Enhancement

**DOI:** 10.3390/s100403023

**Published:** 2010-03-30

**Authors:** Ángel Cuevas Rumín, Manuel Urueña Pascual, Ricardo Romeral Ortega, David Larrabeiti López

**Affiliations:** Department of Telematic Engineering, University Carlos III of Madrid Avda. Universidad, 30. 28911. Leganés-Madrid, Spain; E-Mails: muruenya@it.uc3m.es (M.U.P.); rromeral@it.uc3m.es (R.R.O.); dlarra@it.uc3m.es (D.L.L.)

**Keywords:** survey, data centric storage, wireless sensor network, quadratic adaptive replication

## Abstract

This paper surveys the most relevant works of Data Centric Storage (DCS) for Wireless Sensor Networks. DCS is a research area that covers data dissemination and storage inside an ad-hoc sensor network. In addition, we present a Quadratic Adaptive Replication (QAR) scheme for DCS, which is a more adaptive multi-replication DCS system and outperforms previous proposals in the literature by reducing the overall network traffic that has a direct impact on energy consumption. Finally, we discuss the open research challenges for DCS.

## 1. Introduction

Wireless Sensor Networks (WSNs) have focused the interest of both the industry and academia in the last decade. A wireless sensor is a cheap node with wireless communication capabilities that is able to measure some features of its environment, but has limited storage and battery capacity. When many of these nodes collaborate together to sense some physical phenomenon of interest across a region, transmit it by relay nodes and process it in order to get a common goal, a Wireless Sensor Network is obtained. The main characteristic of WSNs is that their nodes are battery-powered, thus being limited in energy as well as in processing and storage (e.g., memory limited). These limitations have differentiated WSN as an entire research area inside wireless ad hoc networks. Therefore, new protocols and algorithms have been proposed for WSN at all levels of the network stack from physical to application layer [1]. In particular, in [2] we find the main MAC protocols proposals for WSN, whereas the most important routing proposals for WSN are presented in [3, 4]. WSN-specific transport protocols are summarized in [5]. Finally, some examples of WSNs applications are described in [6]. Furthermore, the interest of the industry on WSN has pushed the standardization of light-weight and low power communications technologies that are suitable for sensor nodes. For instance, IEEE specified the 802.15.4 (http://www.ieee802.org/15/pub/TG4.html) standard that covers the physical and MAC layers. On top of 802.15.4, the Zigbee Alliance (http://www.zigbee.org/) has defined a protocol stack covering routing and application layers for WSNs.

One of the most interesting WSN’s research lines is the so called Data-Centric paradigm. The main idea underlying Data-Centric proposals is that for many WSN applications what is important is not who generates the data, but the nature of the data the WSN gathers (also where and when it was obtained could be important). For instance, a node in a sensornet could be interested in establish communication paths only with nodes that measure a given data type (e.g., temperature).

Under this definition some works like Direct Diffusion [7, 8] defined a Data-Centric routing, where routing tables are created in function of nodes interest in particular data types. Therefore, a node forwards a particular message to a neighbour if it is interested in the data contained in the message or it is the next hop towards an interested node.

Besides routing, storage is another widely studied topic in WSN research because the obtained data need to be stored in some way before processing it. There are two straightforward approaches: (i) Local storage, that is, the node measuring the physical phenomenon stores the data. Then, some protocol should be defined in order to allow potential consumers of those data to find and access the nodes where they are stored. (ii) External storage, which means that standard WSN has a central node (sink, base station, etc) that manages the whole network and acts as an access point for external users. Therefore, when a sensor measures a physical event it could push it to the sink that then stores it.

However, a third approach could be used if storage is mixed together with the Data-Centric paradigm. In this approach, a rendezvous node within the sensornet is defined in order to store the information. This alternative storage mechanism is considered a Data-Centric approach when the rendezvous node is selected in function of the data (or event) type instead of a predefined one as in the sink case. With Data Centric Storage (DCS), when a sensor measures a physical event for a particular data type, it pushes the information towards the rendezvous node in charge of that data type. In turn, consumers interested on that data type query that particular rendezvous node to retrieve the information.

Ratnasamy *et al.* [9], introduced the Data Centric Storage term in 2002 as a novel mechanism that disseminates and stores data inside the WSN. This work is the cornerstone of the DCS research field.

Based on this research other authors have studied DCS aspects in order to improve and extend the original proposal. Some of these improvements and extensions are focused on: DCS routing, multi-replication DCS systems that employ several rendezvous nodes, combining DCS with other storage mechanisms depending on network conditions, finding the optimal location to place the rendezvous node, *etc.*

This paper discusses the most relevant works studying DCS in the literature and presents their main contributions. In addition, we introduce in this paper a Quadratic Adaptive Replication (QAR) scheme for multi-replication DCS that is demonstrated to perform better than previous works addressing the same problem. Finally, we discuss future research lines in the area of DCS for WSNs.

Next, we introduce the scenarios where DCS can be applied as well as the terminology used in this paper.

### 1.1. Scenarios for DCS

There are two kinds of sensor networks where DCS could be applied. On one hand, standard WSNs have a central node named sink, base station, gateway, etc, that connects the WSN with the external world. In this kind of networks, sensor nodes gather information from the environment and store it either locally, in a rendezvous node (if DCS is being applied) or push it to the sink that stores all data. In such a network, usually the consumers of the WSN data are external users that query the network through the sink.

On the other hand, nowadays not only sensors but actuators are becoming more relevant in the WSN community, and Wireless Sensor Networks are becoming Wireless Sensor and Actuator Networks (WSANs) [10, 11]. An actuator is a node that, based on the information retrieved from one or more sensors, performs some action. For instance a node controlling a water valve in a plantation opens it depending on the humidity and temperature conditions obtained from the sensors in the field. It must be noted that a node could perform both roles in the network, being a sensor and an actuator at the same time.

The use of WSANs opens up the possibility of autonomous WSANs that, in a distributed way, measure the environment, process the collected data and perform some actions to get a common goal without any external intervention. Therefore, in this case, DCS seems to be a suitable storage and information retrieval system because there is no a clear central, powerful node as in the sink case.

### 1.2. Terminology

This subsection defines some common terminology to be used in the rest of the paper, since different works use different names to refer to the same thing.

- **Event type**: It refers to a physical phenomenon or to some combination of them, providing semantical meaning to the data it refers to. Therefore, an even type could refer to a single physical phenomenon (e.g., temperature, humidity, *etc.*), or to more complex combinations (e.g., an event type ‘FIRE’ is generated when a sensor measures a temperature higher than 100 °C, the smoke detector is over 12 ppm and the humidity has been reduced by a 5% in the last 10 measurements).
- **Rendezvous node or home node**: It is the node responsible of storing the data related to a given event type.
- **Producer node**: When a node detects an event, it forwards the information related to that event towards the suitable home node. Then, we name that node as producer.
- **Consumer node**: It is a node that queries a rendezvous node to retrieve the data related to a given event type.
- **Replica or replication node**: When several nodes play the role of home node for the same data type, we refer to them as replica or replication node.
- **Relay node**: It is a node in the network that does not have any of the previous roles (replica, consumer or producer) so that it is only used to relay messages.

The rest of this paper is organized as follows: The first DCS proposal, GHT, is discussed in Section 2.. Section 3. covers routing proposals for DCS. Section 4. explains the solutions focused on balancing the storage in DCS systems. The main proposals regarding multi-replication DCS systems are presented in Section 5.. Section 6. briefly describes some interesting works about DCS that are related to other topics different than those presented in previous sections. Our Quadratic Adaptive Replication (QAR) scheme for multi-replication DCS systems is described and compared with other proposals in section 7.. Finally, DCS research challenges are discussed in Section 8. that concludes the paper.

## 2. Geographic Hash Table (GHT), the first DCS Proposal

Ratsamany *et al.* [9], first defined the concept of Data Centric Storage (DCS). They combined the idea of Distributed Hash Table (DHT) [12–14] together with Greedy Perimeter Stateless Routing GPSR)[15],a geographic routing protocol, to create a DCS system called Geographic Hash Table (GHT).

This work assumes that sensors are able to locate themselves within the sensornet by using GPS or any other location device or system. In addition, the size and borders of the network are well-known.

**Figure 1.**
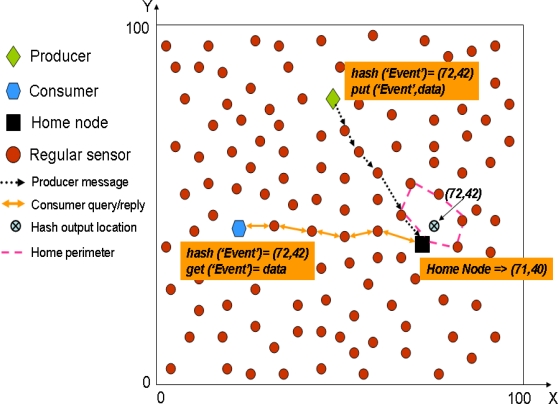
Geographic Hash Table (GHT) example.

In GHT when a producer sensor detects an event, it uses a hash function over the event name (e.g., *hash(’TEMP’)*). The hash function provides as output a spatial location in the sensor field. Then, when a producer detects an event, it gets the spatial location provided by the hash function and invokes a *put(k,d)* operation (where *k* is the key for the event type and *d* the data) that forwards the data towards that spatial location using GPSR. The closest node to that spatial location becomes the home node for that event type and receives the producer message, because GPSR itself is enough to find the closest node to a given position. In turn, when a consumer wants to retrieve the data related to that event type, it uses the same hash function over the event type obtaining exactly the same spatial location. Next, it uses a *get(k)* operation that forwards a query using GPSR to that spatial location, thus reaching the home node that replies with the stored data for that event type. Figure 1 shows a simple example to let the reader better understand how GHT works.

GPSR uses two different algorithms for routing: the first one is called greedy forwarding that in each hop moves the data as closest to the destination as possible, *i.e.*, a node always chooses the closest neighbour to the destination location as the next hop. However sometimes, no neighbour is closer to the destination than the current node, which could be either because the node is already the closest one to the destination, or because it has found a routing hole. In such cases GPSR uses the second routing algorithm, called perimeter forwarding. This algorithm uses the right-hand rule [15] to surround the hole. If during the perimeter forwarding a node closer to the destination that the one starting the perimeter forwarding is found, then the routing is switched again to greedy forwarding. However, if by using perimeter routing the message reaches the same node that started the perimeter routing, that node understands that it is the closest one to the destination coordinates, and therefore the responsible of storing the information related to that event. Then, that node becomes home node and all the nodes in the perimeter that encloses the destination coordinates are called home perimeter.

The authors warn that using a single home node could create a hot-spot problem, therefore they propose to use an additional protocol, called Perimeter Refresh Protocol (PRP), in order to replicate the data in all the nodes of the home perimeter. Thus, reducing the hot-spot of having a single rendezvous node serving all producers and consumers of a given event type. For that purpose, the home node sets up a timer and periodically sends refresh packets along the home perimeter. These refresh packets are directed towards the GHT location provided by the hash function over the event type stored by the home node. In addition, refresh packets contain the data stored for that event type, and they are routed as any other packet in GPSR. Then, when a refresh packet reaches a node there are three options: (i) If that node is closer to the destination coordinates than the home node, then it becomes the new home node. It consumes the refresh packet storing the data it contains and generates a new one. (ii) The node receiving the refresh packet is not closer to the destination coordinates than the home node, then, it just stores the event data and forwards the packet. (iii) If the same refresh packet reaches twice a node that was not the home node, then it becomes the home node. Thus it consumes the packet and establishes its own timer to start generating refresh packets. Therefore, PRP provides fault-tolerance and is able to deal with mobile scenarios. It must be noted that PRP only provides local replication because all the replicas are placed nearby.

In addition, the authors compare three different storage mechanisms: local storage (the producer store the measured data), DCS and external (sink) storage. A simple mathematical model, as well as a deep performance evaluation, are presented indicating when DCS outperforms the other two storage models. GHT focuses on the standard WSN scenario (see Section 1.1.), therefore it assumes that consumption queries are always generated from the sink.

### 2.1. GHT Discussion

One of the main drawbacks of GHT is the complexity added by GPSR when it applies the perimeter routing algorithm, since it requires (among other things) to planarize the connection link graph of the sensornet. Some other geographic routing proposals (e.g., [16, 17]) can be found in the literature and could be also applied to DCS. In addition, there are some proposals that introduce DCS-specific routing protocols (Section 3. covers these proposals).

Using a single home node could create a hot-spot problem because all traffic targets a single node (the home node) that expends its battery quicker than other nodes. The authors propose to use several local replicas, by means of PRP, in order to reduce the potential high load suffered by a single home node. Then, if several replicas are answering consumer queries the load is balanced among them. However, local replication does not solve the routing hot-spot problem. For a very popular content many queries will reach the home perimeter, thus the home perimeter surrounding area becomes a routing hot spot. Therefore local replication helps to balance the home node extra-energy expenditure, but it does not fully solve the routing hot-spot problem. As it will be shown later in this paper, it is necessary to replicate the data in different places along the sensornet in order to reduce that effect (see Section 5.).

## 3. Routing for Data Centric Storage

There are some routing protocols, [18, 19], proposed for WSNs that could be applied to DCS, although they were not specifically designed for this paradigm. Also, there are other proposals in the literature that define novel routing protocols specifically for DCS such as: Rendezvous Regions [20], pathDCS [21] and HVGR [22].

These authors mainly argue that unicast routing using GPSR has several problems, and they focus on other type of approaches. Rendezvous Regions and HVGR are focused on region-oriented routing that does not route messages to a particular spatial location in the network, but to a particular area. In that area there is local knowledge to decide which node becomes the home node. Besides, pathDCS does not map an event type into a spatial location, but to a routing path that ends in the home node.

### 3.1. Rendezvous Regions

This work [20] uses geographic regions as rendezvous areas for producers and consumers. The authors claim that sending messages towards geographic regions instead of geographic points, relax the requirements for location accuracy. In this case an event type is mapped into a region identifier.

The network is divided in rectangular regions where each region could be responsible of one or more event types. The size of a region is several hops, although the exact value is not specified. Each region is assigned with an identifier. Then, each event type is mapped to a region. Finally, each region chooses locally the home node as well as the local replicas.

The authors define 3 different forwarding mechanisms:

- *Unicast*: Direct messages to a given node. This could be used when the location of a rendezvous node is well-known.
- *Geocast*: Sending the message to all nodes in a geographical region. It is useful to send a message to several nodes inside a region (in this case the local replication nodes within that region), however it incurs in an important overhead. The authors propose to use it just for production events.
- *Anycast*: It is enough with reaching one of a set of nodes (one of the local replication nodes). The authors propose to use this forwarding mechanisms for consumption queries.

It is assumed that the nodes are able to detect in which region they are located. In addition, they know how the regions are distributed within the sensornet, that is the region location and its identifier.

Local replicas are elected on-demand. When a production event message reaches the first node in the targeted rendezvous region, this node (known as flooder) geocasts the message inside the region. Each local replica receiving the message sends back an ACK to the flooder. If the ACKs number is not enough (*i.e.*, the number of local replicas is lower than the minimum number established) the flooder geocasts again the message including a self-election probability p. Then, each node that receives the geocast message elects itself as replication node with a probability *p*. This process keeps working until the minimum number of replicas is reached or *p* becomes 1.

When a producer generates an event, it computes the target region and includes its ID within the message. The nodes in the path forward the message towards that region. Once the message reaches the flooder, it geocasts it and receives one (or more) ACKs, that are notified to the producer. If after a given time the producer did not receive any ACK it sends the message again.

This mechanism works similarly for the consumer queries. However, when the query reaches the flooder it uses anycast since it has previously cached all the replication nodes’ location. The selected replica replies to the flooder and the flooder to the consumer.

The authors claim that this work performs better in mobile scenarios than the original GHT. Moreover, the main advantage of this protocol compared to GPSR is that nodes need to know in which region they are located but not its exact position, thus avoiding the necessity of using GPS or any other location device or costly protocol in order to provide each node with its coordinates within the sensor field. On the other hand, the paper neither discuss the size of the area nor how the identifiers should be assigned to each area or how a particular sensor knows which its area identifier is. All these assumptions are key issues in order to practically deploy this routing proposal.

### 3.2. Hierarchical Voronoi Graph Routing (HVGR)

The authors of HVGR [22] follows a similar approach than Rendezvous Regions since they propose to use a region-oriented routing, although, they implement it in a different way. The main contribution of this paper is that it avoids the necessity of each node to use GPS or other location device to know its own location.

The main idea underlaying HVGR is the use of hierarchical coordinates associated with landmarks (nodes whose location is well-known). There is a number of first level landmarks that divide the network into the same number of first level subregions. These landmarks are named *L_i_*, with *i*=1,2,3,...,*m*_1_. Later, inside each first level subregion there are several second level landmarks each one with a different second level identifier. Therefore, inside the subregion belonging to the first level landmark, we find several 2nd level landmarks named as *L*_1,*j*_, with *j*=1,2,3,...,*m*_2_. This division continues until some *n-th* subregion in which all the nodes are within 1 hop distance. Finally, a node is identified as the sequence of landmarks it belongs to, e.g., *L*_1_,*L*_1,1_,*L*_1,1,1_, etc. The number of landmarks in the *i-th* level is defined as *m_i_*.

It is assumed that every node in the sensornet knows all 1st level landmarks. In addition, a node located in a particular 1st level subregion knows the location of all the 2nd level landmarks within that 1st level subregion. In turn, any node in a given 2nd level subregion knows the path to all 3rd level landmarks within that 2nd level subregion, and so on. This knowledge is required to implement the proposed recursive region-oriented routing.

There is a set of hash functions (*H*_1_, *H*_2_, *H*_3_, ..., *H_n_*) employed for routing, each one assigned to a different level. Then, when a producer generates an event, it uses *H*_1_ with the event type and obtains a 1st level landmark to send the message to. However, the message does not necessarily reach that landmark, but the first node within that 1st level subregion receiving the message is able to route the message towards the suitable 2nd level landmark. In order to do this, that node uses *H*_2_ over the event type and obtains the 2nd level landmark. Following this routing mechanism the message finally reaches the rendezvous node in charge of that event type.

HVGR requires a first phase where the hierarchical infrastructure is built, which means creating the region and selecting the landmarks. There is a first node, called 𝒞, which starts the landmark selection process, and it is defined as the master among all the first-level landmarks. HVGR uses [23] to select all the *m*_1_ 1st level landmarks. Each of these 1st level landmarks is the master landmark within its 2nd level subregion, and they repeat the operation to find the rest of *m*_2_ − 1 landmarks in each 2nd level subregion. The process stops when a master landmark detects that all the nodes within its region are their direct neighbours. It must be noted that, by using this algorithm, a 1st level landmark is also a landmark for the rest of the levels.

Once landmarks have been selected, there is an initial process in which every node in the sensornet learns the landmark coordinates. All the 1st level landmark nodes flood a LANDMARK message, so that each node in the network learns the path to all the 1st level landmarks. Next, each node selects the closest landmark. In such way, the network is divided into *m*_1_ first level subregions. Following, the 2nd level landmarks make a scooped flood within its 1st level region. Again, a node knows all the 2nd level landmarks within its 2nd level subregion, and the 2nd level subregions are generated when each node selects its closest 2nd level landmark. By running this recursive algorithm the network is ready to employ the region oriented routing.

This solution relies on a hierarchical infrastructure that avoids each node to know its own spatial location. Instead of this, each node knows the path to a set of landmarks and route the messages towards them. This routing is demonstrated to be more efficient that GPSR. The disadvantage is the cost related to the hierarchical infrastructure creation. Choosing the landmarks as well as notifying their position to the nodes implies to use quite a lot of messages, in particular for large WSNs.

### 3.3. PathDCS

PathDCS [21] also relies on landmarks, here called beacons, to route messages and queries avoiding the necessity of point-to-point routing (such as geographic routing) that requires location knowledge.

The authors indicate that point-to-point routing presents problems in WSNs, so that is better to define tree-structures in order to route messages. In particular, pathDCS creates one tree per beacon node, rooted at the beacon node. They use existing algorithms for the tree creation [24, 25] and use [26] to elect the beacon nodes.

Each node is assigned with a logical identifier. The authors remark that in DCS, based on a key (event type), *k*, the home node should be consistent for any production event or consumption query. Then, the novelty of this proposal is that an event type is not mapped to a spatial location, but to a path. Roughly speaking, based on the event type there will be a set of indications: ”you have to go to beacon 1, later you have to travel two hops towards beacon 2 and finally 1 hop towards beacon 3”. Therefore a path consists of a sequence of *p* beacons *b_i_*, and lengths *l_i_*, with i=1,2,...,*p*. Then, an event (production) or query (consumption) is sent to beacon *b*_1_ (using the tree created by *b*_1_), next it is forwarded *l*_2_ hops towards beacon *b*_2_, and so on, until it is sent from the last node *l_i_* hops towards *b_i_*, where *l*_1_ is the distance in hops between the source node and the beacon *b*_1_.

A beacon node *b_i_* is the node whose identifier is the closest one to *hash(k,i)*, whereas the length of the *i-th* segment is obtained as: *l_i_* = *h*(*k, i*) *mod hops*(*n_i_, b_i_*), where *n_i_* is the node that starts the *i-th* segment and *hops(n,b)* the distance in number of hops between the node *n* and the beacon *b*. Therefore, in order to apply this proposal two parameters are required, the number of beacon nodes within the network and the number of segments used in a path, *p*.

Moreover, they also propose to use local replication in the k-hops home node’s neighbours to reduce the load of the home node, which is a local replication mechanism.

PathDCS is a very interesting approach that has been implemented and tested in real sensor nodes. As HVGR, it relies on an infrastructure that provides references to all the nodes within the sensornet to perform the routing without knowing its own spatial location, but this seems simpler than the HVGR one. The main disadvantage is that pathDCS needs to create one routing tree per beacon node, which implies additional energy consumption. The energy for creating the beacon infrastructure depends on the size of the WSN and the number of beacon nodes used.

## 4. Balancing Storage in DCS Systems

The home node (single DCS) or the replication nodes (multi-replication DCS) are the nodes that store the data events on behalf of producers. In many applications data need to be accessible during certain time, thus home nodes could suffer a higher memory utilization. Therefore, at some point the memory of a home node could be saturated, and some mechanisms are required either to avoid this situation or to select a new home node when it is overloaded. In addition, in heterogeneous WSN where some nodes have more memory than others, it would be interesting to select those ones as home nodes.

This section presents two relevant works [27, 28] that are focused on balancing storage in DCS systems.

### 4.1. Dynamic DCS

The authors of [27] propose to use a dynamic GHT that is based on two main features: (i) A temporal-based GHT, that changes the home node over the time. (ii) A location selection scheme that chooses as potential home nodes those with more memory and battery resources. They say that with these two improvements the sensornet lifetime will be significantly extended.

The authors focus the problem in node storage capacity, indicating that the home node of an event type could run out of memory, so that it could not be able to store new events. For that, they divide the network into grid cells. First of all they assume that there is one home node per event type that is allocated in the center of a cell. Then, they propose a hash function that permits allocating the home nodes of different event type in different cells. In addition, this hash function has into account a time slot Δ*t*, to allow the home node of a given event type changes over the time. Both the event types and the cells are assigned with a numerical identifier. Then, the hash function uses as input the time slot number (*t*) and the event type identifier (*e_i_*), and gives as output the cell number identifier where the home node will be placed for that event type. Some other required values are: the total number of cells (*n*) and the total number of events (*e*), because in the formula is used the least common multiple (*M*) of *n* and *e*. In particular a parameter *ϑ* = *M/e* appears in the formula. The proposed hash function is :

$$h\left(e_{i},t\right)=\left(\mathrm{te}+i-(t\;\mathrm{div}\;\vartheta )\right)\;\mathrm{mod}\;n$$

The best way of understanding how it works is with an example. Let us think in a network with 100 cells (*n*) and 10 event types (*e*). Then, being *M* = 100, *ϑ* = *M/e* = 10. Table 1 shows the cell number associated with a particular event type for different time slot numbers.

It can be appreciated that a home node for a particular event type remains in the same cell during *ϑ* time slots. After that time, it moves to the cell with the immediately lower identifier.

In addition, the authors try to solve the problem of selecting the node with more resources in a particular cell as the home node. They use the Node Contribution Potential [29] as a measurement of the node capacity. In particular, this value is a combination of the storage capacity of the node and its remaining battery. Then, all the nodes inside a cell exchange their contribution potentials in order to calculate the cell potential, which is sent to the sink together with the location of the node with higher contribution potential within the cell. In turn, the sink selects a set of cells above a given cell potential threshold to be the location set for an arbitrarily long period, and broadcasts this set, together with the coordinates associated with it. During that period all nodes use that location set. Therefore, being *n_L_* the number of cells in the set, the new hash function is:

$$h\left(e_{i},t\right)=(\mathrm{te}+i-(t\;\mathrm{div}\;\vartheta ))\;\mathrm{mod}\;n_{L}$$

This paper enables changing the home node for a given event type over the time. Thus, the authors claim that this alleviates the hot spot storage problem. However, they do not explain what happens with the already stored data during the transition from the old home node to the new one, thus it seems that the data stored in the old home node is lost. Furthermore, practical issues like the number of cells to be used, how they are assigned with an identifier, and how the sensors are able to know the identifiers of each cell, are not discussed.

### 4.2. A grid-based dynamic load balancing approach for DCS

This recent work [28] also applies the idea that when a home node becomes saturated, a new one needs to be elected. The main difference between this work and the previous one [27], is that the former selects a new home node to store the new production events, but still keeps the previous one since now consumer queries will get data from both, the old and the new home node, whereas [27] just assumes that after a given period a new home node is selected, but it does not discuss what happens with the data stored in the old home node. The authors in [28] divide the network in a grid with cells of the same size in such a way that they assure that all the nodes inside a cell are within one hop distance. Then, each grid is assigned with positive coordinates (*X*_*grid*_*i*__, *Y*_*grid*_*i*__) called *grid ID*, which are based on its spatial location. Each sensor, given its own coordinates (*x_i_, y_i_*), is able to calculate its own grid ID. Therefore, each sensor knows its grid ID, its own coordinates and its grid neighbours coordinates. In addition, each node also has and share with its neighbours a virtual grid ID and virtual coordinates, which initially are equal to the actual grid ID and actual coordinates respectively.

Then when a producer detects an event of a given event type, it calculates the home grid ID (no the spatial coordinates whose closest node is the home node) by means of a hash function. Then, once the production message reaches the first node in the home grid, it sends the message to the other grid neighbours, that are only 1 hop far away. Since, all nodes know their neighbours’ coordinates, they compute who is the closest one to the center of the grid, which becomes the home node for that event type. The remaining nodes in the grid just discard the message. They use the virtual coordinates already explained for this computation.

Next, the authors define several storage thresholds, *i.e.*, the first storage level is *S*_1_ events (e.g., 30 events), the second, *S*_2_ events (e.g., 60 events), until a maximum threshold that is the maximum storage capacity. Once the home node reaches the first storage level, it changes its virtual coordinates to (∞,∞) and sends this update to its grid neighbours. Therefore, following the operational mode presented in the paper, the second closest node to the center of the grid becomes the new home node from that moment. It is possible, that at some point all the nodes reach the 1st level threshold. The last node reaching this threshold (the furthest one from the center of the grid) notifies this fact. Then the 2nd storage threshold is established in the grid, and all the nodes within the grid reset their virtual coordinates being again the same than their actual coordinates. However, following this mechanism at some point all the nodes within a grid could be saturated (reaching the last storage threshold). In such a case the authors propose something called extended grid, that is, using all the adjacent grids to the saturated one to select a new home node.

Finally, when a consumption query reaches the grid, it is received by all the nodes in the grid. Therefore, all the nodes with some stored data for the queried event type replies back to the consumer, which in this paper is always the sink.

This proposal solves in a smart manner the storage problem by selecting a new home node when the previous ones reach an established storage threshold. This solution assumes that the network is divided in areas of the same size so that all nodes in an area are in range of each other. However, with this definition there would be many overlapped areas or nodes that could belong to several areas. Therefore, the definition of how the sensornet is partitioned should be more precise.

## 5. Multi-Replication in Data Centric Storage

It has been already mentioned that one of the main drawbacks of the original Data Centric Storage (DCS) proposal is the hot-spot creation when a single node is in charge of a popular event. Local replication (using nearby nodes as replicas) does not fully solve the problem but just mitigate it, since still all traffic will be directed to a well defined area, which becomes a hot-spot zone.

Therefore several works in the literature propose to use several replication nodes allocated along the network in order to avoid the hot-spot area problem. These works proposes different replication structures: grid [30–33], random [34], circumference [35] and mesh [36–41]. Next, this section covers the most important works proposing multi-replication in DCS systems.

### 5.1. GHT with multiple replicas

**Figure 2.**
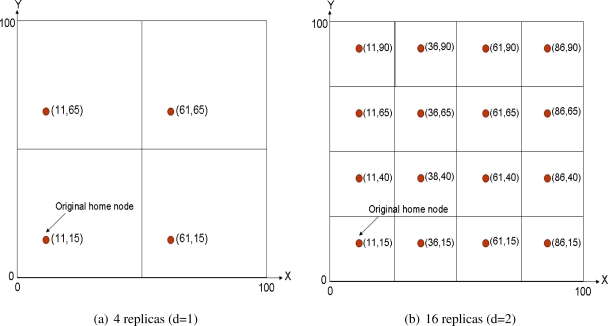
Multi-replication grid in GHT .

Ratsamany *et al.*, the original DCS authors, were already aware of this problem and proposed a grid-structured multi-replication system [30, 31]. They assume a square sensornet field that is divided into 4*^d^* grids, being *d* the so-called network depth. The original home node is established as defined in GHT, that is applying a hash function over the event type. After that, 4*^d^* − 1 mirror replicas are allocated in the same relative position inside each cell of the grid. Figure 2(a) shows a multi-replication scenario with *d* = 1 and Figure 2(b) with *d* = 2. Of course the coordinates shown in the figures refer just to space locations, thus the closest node to each location is the home replica node in that grid. This mechanism is recursive, because replicas of level *d* are also replicas of level *d* + 1.

**Figure 3.**
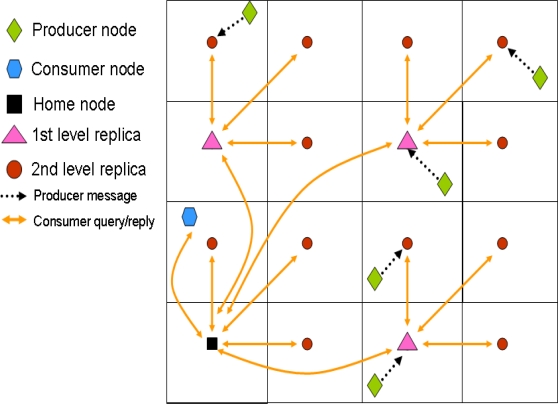
GHT multi-replication scenario.

With this system, a producer generating an event stores it in the closest replication node. Then, in order to retrieve all the data related to a particular event, a consumer queries the top home node (*d* = 0). This one in turn forwards the query to the remaining first level replicas (*d* = 1), that in turn do the same with their second level replicas (*d* = 2), and so on, in a recursive manner. The replies to the query travel the same path back to the original home node, which finally sends the information back to the consumer. Therefore, the information retrieval is based on a hierarchical routing that also relies on GPSR. Figure 3 depicts an scenario example including both production and consumption communications.

There are no further discussion of multi-replication in [30, 31], but just a final remark: the proposed muti-replication environment reduces the storage cost in front of using a single home node, but increases the query cost, since now consumer packets travel longer to get the information.

This work is the first one introducing the possibility of using several home nodes instead of only one. However, the authors do not provide any way to determine a suitable *d* in order to minimize the query cost.

### 5.2. Resilient Data-Centric Storage

In this work [33], a two-level replication strategy is proposed for control and data. The sensornet is divided into *Z* zones. Each zone could contain 3 types of nodes:

- **Monitor Mode**: Each zone has one monitor node for each event type. The monitor nodes communicate to each other by exchanging information of monitor maps that includes control and summary information. The information contained in this monitor maps are: list of zones containing replica nodes, list of zones containing monitor nodes, event summaries and bloom filters that are used for attribute-based queries (i.e. get temperatures over 30 Celsius degrees).
- **Replica Mode**: Each zone has at most one replica node for each event type. In addition, if the replica node is present, it is the same node than the monitor node. The extra function is to store event data for the given event type.
- **Normal Mode**: These are nodes that are neither monitor nor replica nodes.

The storage model is quite similar to the one proposed in GHT with multiple replicas: a node that generates an event forwards it to the monitor node in its zone. If the monitor node is a replica of that event, it stores the data, otherwise, since it has the list of zones containing replica nodes, forwards the data to the closest replica.

The main contribution of this paper is the different query types that proposed, which are more sophisticated than the one proposed in [30, 31]. There are three types of queries:

- **List**: This is the one described in GHT [30, 31]. A consumer sends a query requesting the data related to an event type to the monitor node in its area. This one forwards the query to all the replication nodes for that event type. Then, they reply directly to the consumer node with all the requested data.
- **Summary**: The consumer requests a summary of the event type information. It contacts its local monitor node that replies directly with the summary for that event type (e.g., average, max and min value of that event type).
- **Attribute-based**: It is a query requesting data for different events which match certain constraints in the attribute values. For that purpose bloom filters are used since they provide a compact summary of data without false negative. A consumer sends a query to its local monitor node for a given event type. Then the monitor node duplicates the query and sends it to all the replication nodes with bloom filters matches. All the replicas reply directly to the querying node.

This work extends and complements the top level of a multi-replication DCS system like GHT with multiple replicas, but it also adds complexity since an extra role is included in the system, the monitor node, which implies more energy expenditures for that task. As previous proposals, this work does not provide any discussion about the appropriate number of zones, *Z*, to be used.

### 5.3. Tug of War (ToW)

None of the previous works study how many replicas should be allocated in a multi-replication DCS system depending on network conditions for a given event type.

ToW [32] is, to the best of our knowledge, the only work in the literature that has addressed this problem. ToW provides the number of replicas that should be used following the 4*^d^* schema introduced by multi-replication GHT [30, 31] in order to minimize the overall network traffic.

**Figure 4.**
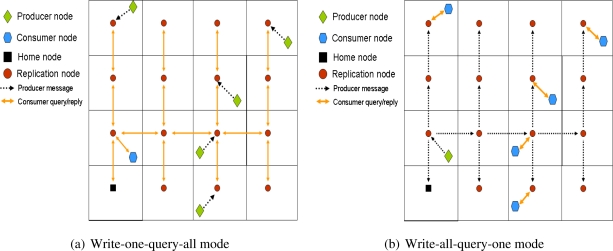
Multi-replication grid in ToW with combing routing.

ToW presents 3 new contributions to DCS multi-replication research:

1. It provides an analytical model that computes the optimal value of the network depth, *d*, in order to minimize the network traffic.
2. It describes two different DCS operation modes:
  - **Write-one-query-all**: This is the same mechanism introduced in GHT with multiple replicas. Producers store the information in its closest replica. Then a consumer query has to reach all replicas in order to retrieve all the information related to an event type. An example of this operation mode is shown in Figure 4(a).
  - **Write-all-query-one**: It is a novel mechanism in which producers store the data in the closest replica, that in turn forwards the information to the remaining replicas. Therefore, all the information related to a particular event type is stored in every single replica. Thus, a consumer only needs to query its closest replication node to retrieve all the information. Figure 4(b) shows an example of this operation mode.
3. It proposes a new routing mechanism that is not hierarchical and recursive like the one proposed by multi-replication GHT. Since replicas are placed in a grid, the authors propose to connect them using a grid replication tree and call it “combing routing”. Therefore, when a replica receives some data from a producer, in the write-all-query-one mode, it forwards the information along its own row and its own column. The replicas belonging to the same row replicates the information along its column (see Figure 4(b)). In the case a replica receives a query from a consumer in the write-one-query-all mode, it follows the same strategy, the replica routes the query to the rest of the replicas using the combing routing. In this case the replies follow the same path back (see Figure 4(a)). This means (in write-one-query-all mode) that some kind of data aggregation [42] must be in place in order to deliver replies efficiently, although the authors do not study this issue.

ToW is analytically compared with multi-replication GHT, and it is shown to be more efficient due to the utilization of the combing routing instead of the hierarchical-recursive routing proposed by GHT with multiple replicas.

Moreover, ToW proposes an analytical model to define the optimal value of the network depth, *d* (which leads to the number of replicas to be used). The authors analyze a scenario where *n* sensors are uniformly deployed in a square region, thus each side of that region has roughly $\sqrt{n}$ nodes. The authors call *f_e_* and *f_q_* the frequency of producers’ events and consumers’ queries respectively. In addition, they define a parameter *k* that denotes the average number of sensors that detect the same event.

The distance between two random nodes inside a unit square is (http://mathworld.wolfram.com/SquareLinePicking.html), *δ̄*= 0.52. This helps to model what is the distance from a consumer or producer to its closest replica.

#### Write-all-query-one Mode

The measured cost (in distance) for a consumer to send a query to its closest replica with *d* = 0 has a cost of $\bar{\delta }\sqrt{n}$. If *d* increases, then the distance to the closest replica is divided by a factor 2*^d^*. Therefore, the consumer querying cost per unit interval could be defined as

$$C_{qW_{\mathrm{all}}Q_{1}}=f_{q}\frac{\bar{\delta }\sqrt{n}}{2^{d}}$$

Later, the cost of storing an event in all the replicas (overall producers’ event cost per unit interval) allocated for that event type is:

$$C_{eW_{\mathrm{all}}Q_{1}}=f_{e}k\frac{\bar{\delta }\sqrt{n}}{2^{d}}+f_{e}k\left(4^{d}-1\right)\frac{\sqrt{n}}{2^{d}}=f_{e}k\sqrt{n}\left(2^{d}-\frac{1-\bar{\delta }}{2^{d}}\right)$$

It is composed by two terms: (i) the first one refers to the cost of forwarding the data from the producer to its closest replica, (ii) the second cost is due to replicate the received data in all the remaining replicas using combing routing. Since we are assuming a uniform deployment, the distance between any two replicas is the same, $\frac{\sqrt{n}}{2^{d}}$. In order to compute the total cost it is enough with knowing the number of branches used to replicate the information, that is, 4^*d*^ – 1.

Therefore, the total cost for the write-all-query-one mode is:

$$C_{W_{\mathrm{all}}Q_{1}}=C_{eW_{\mathrm{all}}Q_{1}}+2C_{qW_{\mathrm{all}}Q_{1}}=f_{e}k\sqrt{n}\left(2^{d}-\frac{1-\bar{\delta }}{2^{d}}\right)+2f_{q}\frac{\bar{\delta }\sqrt{n}}{2^{d}}$$

Notice that the querying cost is multiplied by a 2 factor so that the cost of the reply to the consumer query is also computed.

The optimal value of *d* in order to reduce the overall cost in the network is obtained by performing an optimization of the previous equation in function of *d*. Therefore, this optimal value, d*, is:

$$d_{W_{\mathrm{all}}Q_{1}}^{*}=\frac{1}{2}\log\left(\frac{2\bar{\delta }}{k}\frac{f_{q}}{f_{e}}-\left(1-\bar{\delta }\right)\right)$$

#### Write-one-query-all Mode

In this case the cost of storing an event (production cost) in the closest replica is immediate:

$$C_{eW_{1}Q_{\mathrm{all}}}=f_{e}k\frac{\bar{\delta }\sqrt{n}}{2^{d}}$$

The authors also use a symmetric cost model for the consumption cost, and they define it as:

$$C_{q_{W_{1}Q_{\mathrm{all}}}}=f_{q}\sqrt{n}\left(2^{d}-\frac{1-\bar{\delta }}{2^{d}}\right)$$

Therefore, the overall cost and d* are:

$$\begin{array}{c} C_{W_{1}Q_{\mathrm{all}}}=C_{eW_{\mathrm{all}}Q_{1}}+2C_{qW_{\mathrm{all}}Q_{1}}=f_{e}k\frac{\bar{\delta }\sqrt{n}}{2^{d}}+2f_{q}\sqrt{n}\left(2^{d}-\frac{1-\bar{\delta }}{2^{d}}\right)\\ d_{W_{\mathrm{all}}Q_{1}}^{*}=\frac{1}{2}\log\left(\frac{\bar{\delta }k}{2}\frac{f_{e}}{f_{q}}-\left(1-\bar{\delta }\right)\right) \end{array}$$

As it has been already mentioned, this model is symmetric to the write-all-query-one model. This is only possible if aggregation is assumed in the query reply path. Therefore, the distance cost of a query is the same than that of its reply.

In order to apply this model, regular sensors need to know two parameters: *k* and the $\frac{fq}{fe}$ ratio. For *k*, the number of sensors measuring the same event, the authors do not present any discussion and assume that is a well-know value in the network. However, in a real WSN calculating the actual *k* value is quite complex and in general it will not be homogeneous across the network. For the $\frac{fq}{fe}$ ratio, replication nodes are in contact among them, therefore they have a global overview of this ratio in the network. Then, the ratio is attached to each message sent along the consumption reply path, so those nodes that are in the range of the replying path could learn it. In addition, each regular node includes this ratio when it forwards any message. However, this mechanism cannot assure that all producers and consumers are aware of the current $\frac{fq}{fe}$ ratio, which could lead to a worse performance since some sensors could evaluate an incorrect number of replicas.

### 5.4. Double Rulings for Information Brokerage in Sensor Networks

Previous works employ structured grid-based replication. This work [35] is a much more complex approach that uses circumferences to replicate the data. The basic idea is: a producer generates a big circumference between itself and the home node, storing the information in all the nodes along the path. A consumer for that event type does exactly the same, so that, at some point, this two big circumferences crosses each other (at least in the home node). Thus, the node located in the crossing can reply to the consumer with the data.

It is obvious that all the production curves related to a given event type have at least a common node, which is the home node. However, the paper demonstrates that there is a second point where all the production circumferences for a given event type collide. Therefore, there are at least two nodes that store all the events of a given type, meaning two global replication nodes (these are always two in contrast with multi-replication GHT and ToW that can adapt that number).

Another contribution of this paper is that it defines several ways to retrieve the data depending on the application’s interest:

- **GHT retrieval**: A consumer accesses one of the two home nodes (the closest one) and retrieve all the information.
- **Distance-Sensitive retrieval**: It defines a mechanism to assure an upper bound distance when a consumer wants to retrieve the data from a particular producer, then the consumer has to find the cross between its consumption circumference and the suitable production circumference.
- **Aggregated data retrieval**: The consumer uses a closed curve that separates both home nodes, collects all relevant data and computes the aggregates.
- **Double rulings retrieval**: It defines the full power data retrieval rule. That is, the consumer travels along any great circle and is able to retrieve all the data stored in the sensornet.

The authors compares its proposal in front of GHT without replication [9] and GHT with replication [30, 31] being the network depth 1 (*d*=1). Table 2 shows the average producer and consumer costs in hop numbers for the three approaches. The authors conclude that double rulings clearly outperforms GHT without and with replication. However, this may be not true in all the cases, since it very much depends on the number of producers’ events and consumers’ queries. For instance in a scenario with many producers’ events and just few consumers’ queries, GHT without replication would provide better results than Double Rulings just by following the data provided in the paper.

The main drawback of this paper is the complexity of implementing the proposed routing. Forwarding messages following a curve is far from being the standard routing mechanism and surely adds complexity to the implementation. Furthermore, as it has been already mentioned, Double Rulings can only employ 2 global replicas, which could be not enough in some scenarios, whereas other solutions like ToW or GHT with multiple replicas could adapt the number of replicas to the existing traffic load.

### 5.5. Mesh Replication Proposals

There are some works in the literature [36–41] that can be seen as an alternative to DCS systems. In this section we provide a brief overview of the basic idea, to let the reader understand how these systems work.

**Figure 5.**
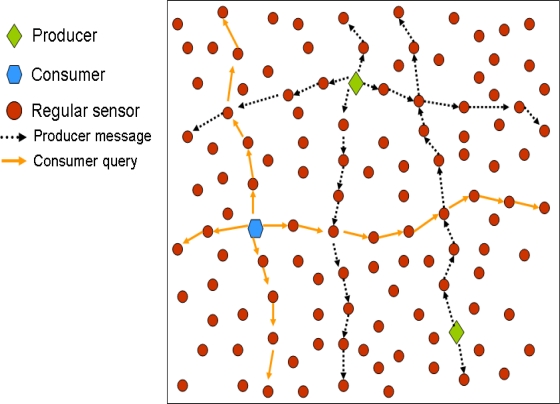
Mesh Replication.

A producer that has measured an event, forwards a message in the North (N), South (S), East (E) and West (W) direction, and all the nodes in these paths store the data. In turn, a consumer that wants to retrieve this data, also sends the query in the North, South, East and West directions, so that it is sure that the query will cross at least one of the nodes storing the data of interest. In addition, if there are several producers of a given data type, the consumer will be able to retrieve all the data with just one query. Figure 5 shows an example of these proposals.

The authors in [36] present a mathematical analysis and a deep performance evaluation via simulation of this mechanism. A proposal to find the position of a mobile node within the network is studied in [37, 38] and [41]. The authors propose that the mobile node updates its location sending a message in all the directions, so that all the nodes in those paths store the current position of that particular mobile node. Then, a node that wants to find the current position of the mobile node sends a also query in the four directions (E, W, S and N). In [40], a distance-sensitive service discovery is proposed based on the mesh replication structure. Finally, the authors in [39] use the proposed scheme and present a Mesh-based Sensor Relocation protocol (MSRP) for mobile sensor networks. This protocol maintains the sensornet sensing coverage by replacing failing nodes with others that are close by.

## 6. Other issues

Previous sections have covered the main issues related to Data-Centric Storage: multi-replication, routing and storage for DCS systems. However, there are more works in the literature that focus in different aspects of DCS. This section briefly introduces the most relevant ones.

In [43] the authors try to find the optimal home node position based on the event production and query consumption distributions. They define a mathematical model in order to solve the problem. However, the implementation of the defined algorithm is not distributed, but centralized, since the base station calculates the optimal home node location. For that purpose, it is required that the base station has full knowledge of event and query distributions for a given event type, that initially is something that is not required to in a DCS system.

There are several works [44–47] that compare the different storage policies and analyzes which is the suitable relation between queries and events to apply each storage mode.

A new storage mode (different than local storage, DCS and external storage) is introduced in [44] and later used in [45] and [46]. This mode stores the data in a node close to the producer (or locally in the producer) and notifies this to the home node. The home node has an index list with all the producers storing data for a given event type. Therefore, when it receives a consumer query, it retrieves the data from all the producers in the list and replies back to the consumer. In all these works the queries are generated from the sink.

The author in [47] proposes an adaptive storage policy switching between local storage and data-centric storage depending on the event-production to query-consumption ratio. In this case, the queries are generated from several mobile sinks within the sensornet. In addition, the network is divided into multiple grids, and each grid decides locally whether to apply data-centric storage or local storage. The author describes the full communication model as well as the policies to switch from one storage mode to the other. This work assumes that each grid is assigned with an identifier and the messages are sent to a particular grid based on that identifier. In addition, it is also assumed that each sensor knows to which grid it belongs.

There are some works [48, 49] that employ DCS to support multi-dimensional data queries in WSN. In order to achieve that goal, they create a *K-D tree* where nodes are assigned with bit-code identifiers that are related to their spatial location in the network. Then they split the events value range and assigns a bit code to a particular event type sub-range of values. Therefore, the multi-range queries are generated by a sequence of different event type bit codes. This sequence results in another bit code that is sent to the node with the closest bit-code identifier, thus creating the DCS system.

Finally, there are a set of works Q-NiGHT [50], DELiGHT [51] and [52] that propose to use a novel hash function based on a rejection method [53]. This function is aware of the sensor distribution and avoids to provide a home node location in areas with very low node density (e.g., borders of the network). Furthermore, these works add QoS to DCS. The authors claim that depending on the event type, the number of local replicas should be different. Then, when a producer pushes an event towards the home node, it includes a *Q* parameter that indicates the number of local replicas to be used. In turn, the home node chooses the replicas from a surrounding area. Q-NiGHT and DELiGHT propose to replicate each event in all the replication nodes, whereas [52] applies erasure codes [54] that codify the data in smaller pieces, storing each one in different replicas. Therefore, in order to reconstruct the information, a consumer accesses the replicas retrieving all the codified data and reconstructs the event data. This mechanism better spreads the storage load among the replicas.

## 7. Quadratic Adaptive Replication (QAR)

Multi-replication is one of the most important research lines of DCS. In particular, ToW [32] is one of the most interesting works because it is the only one that has presented a model that provides the optimal number of replicas to be used based on network traffic. ToW is based on a geometric replication formula, that calculates the number of replicas, *N_r_* = 4*^d^*, being *d* the so called network-depth. Once the optimal *d*, d*, has been defined, the number of replicas to be used is also known. The main drawback of this replication scheme (that is also used in GHT with multiple replicas [30, 31]) is that it is not very flexible because the number of replicas could be only one among these values: 1, 4, 16, 64, 256, 512, 1,024, *etc*. Therefore, there could be cases where, for instance, neither 16 replicas nor 64 are the optimal value, but some other value in between.

**Figure 6.**
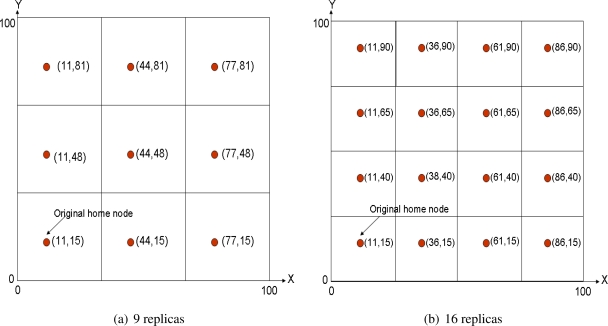
Multi-replication grid in Quadratic Adaptive Replication (QAR).

In order to provide a more flexible solution that allows to select a more adaptive number of replicas, we have designed the Quadratic Adaptive Replication (QAR) scheme, where the number of replicas, *N_r_*, follows a quadratic formula, *d*^2^. QAR is also a grid-based replication scheme that uses the combing routing used in ToW (explained in section 5.3.). QAR is demonstrated to be much more adaptive than ToW, since now the number of replicas that can be selected is: 1, 4, 9, 16, 25, 36, 49, 64, 81, 100, 121, 144, *etc*. Figure 6 shows an example of QAR for *N_r_* = 9 and 16. It must be noted that ToW does not have the option of using 9 replicas, but jumps from 4 to 16.

We also present an alternative model to the one of ToW that provides the optimal number of replicas to be used in order to minimize the overall network traffic. Our model considers the same cases proposed in ToW: when the traffic related to queries overpass the one generated by events (“consumption dominates production”) and the opposite case (“production dominates consumption”). These cases are the same described in ToW as “write-all-query-one” and “write-one-query all”, respectively.

Therefore, when there are many consumer queries (consumption dominates production), it is worthy to replicate the few producer events in all replicas using the combing routing, while consumers only need to access the closest replication node in order to retrieve all the data. On the other hand, when there are few consumers queries (production dominates consumption), replicating all data as in the previous case is quite inefficient. Instead, in this case producers just store the events in its closest replica while consumer queries need to reach all replication nodes using the combing routing in order to retrieve all the data of an event type.

Finally, it must be noted that our mathematical model provides an optimal number of replicas that is a real number (this also happens in the ToW model). Thus, we evaluate the overall traffic cost for the immediate higher and lower integer number obtained from the quadratic series 1, 4, 9, 16, 25 *etc.* For instance if the model output is $N_{r}^{*}=29.31$, we evaluate the traffic cost with *N_r_* = 25 and *N_r_* = 36 and select as number of replicas the one providing the lower traffic cost. It must be noted that in the same case ToW could only choose among *N_r_* = 16 or *N_r_* = 64.

### 7.1. Analytical Model

In this section we present our analytical model that will enable us to establish the optimal number of replicas so as to minimize the overall network traffic.

We shall start by developing a model for the overall network traffic generated by consumption and production nodes across the network. The overall metric is the total traffic load, measured in m-bits/sec that is supported by the network, to take into account both the data rate and the traveled distance across the WSN.

The locations of nodes of the wireless sensor network are fixed and modeled by a homogeneous spatial Poisson Point Process Π*_n_* [55], *i.e.*, a random set of points on the plane, with intensity λ*_n_* nodes per unit area. Production and consumption events generated by network nodes are in turn modeled via independent homogeneous spatio-temporal Poisson Point Processes Π*_p_* and Π*_c_* each with intensities *λ_p_* and *λ_c_* events per unit time and unit area respectively. There are *λ_r_* nodes per unit area placed in a grid fashion serving as replication nodes. Although the model corresponds to one on an infinite plane, we shall restrict attention to a fixed region 𝒜 ⊂ ℝ^2^ modeled as a convex set with area *A* = |𝒜|, and then optimize operation on 𝒜, roughly ignoring edge effects. On average there are *N_r_* = *λ_r_A* replication nodes in 𝒜. Thus, a quadratic hierarchy level *d* could be defined as, $d=\sqrt{N_{r}}$.

#### Consumption dominates Production (*λ_c_ > λ_p_*)

When consumption dominates production, consumers retrieve data from the closest replication node. Then the average distance between a node and its closest replica is roughly the average distance between two nodes inside one of the replica’s square cells. This distance can be modeled as $\frac{\delta }{\sqrt{\lambda_{r}}}$ with *δ* = 0.52 (the same used in ToW). Taking into account that the total number of consumption events in a sensornet of area *A*, is *λ_c_A*, the next expression defines the overall network traffic associated with consumption traffic (*T_c_*),

$$T_{c}\left(\lambda_{r}\right)=\alpha \lambda_{c}A\frac{\delta }{\sqrt{\lambda_{r}}}\;\;\text{bits-m}/\text{sec},$$

where *α* is a proportionality constant corresponding to the average number of bits per consumption event that are exchanged between the consumer and its nearest replication node.

Next we consider the replication cost when data is produced. This cost is composed of two different factors: the first one is due to a producer sending information to its closest replica and the second cost to the replication process from that closest replica to the remaining replication nodes.

Therefore, the former cost is calculated in the same way than the consumption cost. The latter needs to consider all the branches in the generated replication tree. Since we rely on a grid-replication structure, the length of all the branches is exactly the same. It is the distance between the center of two neighbour square cells, that is, the distance of one side of the square cell, which is $\frac{1}{\sqrt{\lambda_{r}}}$ in a square-unit area.

The overall production traffic in a square WSN field of area *A*, is composed of : (i) the number of branches to be traversed by the replicated information, which is the number of replicas minus 1, thus *λ_r_A* − 1 (*N_r_* = *λ_r_A*); and, (ii) the number of production events in the sensornet, *λ_p_A*. Thus, the overall production traffic is

$$T_{p}\left(\lambda_{r}\right)=\beta \lambda_{p}A\frac{\delta }{\sqrt{\lambda_{r}}}+\beta \lambda_{p}A\left(\lambda_{r}A-1\right)\frac{1}{\sqrt{\lambda_{r}}}\;\;\;\text{bits-m}/\text{sec},$$

where *β* plays the same role for production cost than *α* in the consumption cost, that is, the average size in bits of the production event messages.

Thus, the overall network traffic is computed as *T_t_*(*λ_r_*) = *T_c_*(*λ_r_*) + *T_p_*(*λ_r_*). By optimizing this expression we are able to find the optimal number of replicas ( $N_{r}^{*}$) in order to minimize the overall network traffic,

$$N_{r}^{*}=\lambda_{r}^{*}A=\delta \frac{\alpha \lambda_{c}}{\beta \lambda_{p}}-(1-\delta )$$

**Figure 7.**
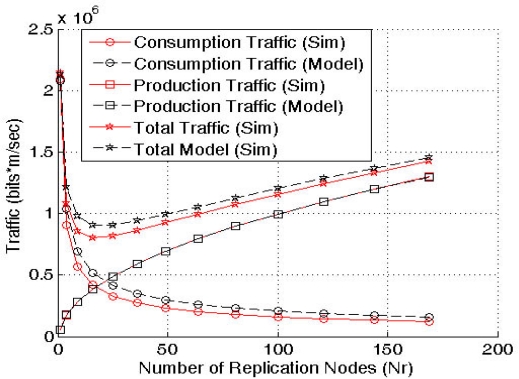
Overall network traffic generated by QAR using different number of replicas (A = 1,000 × 1,000, N = 5,000, *α* = 200 bits, *β* = 100 bits, *N_p_* = 100; *N_c_* = 2000, Iterations = 100).

Figure 7 shows the model validation via simulation. The results demonstrate that the model is accurate enough and can be used to find the optimal number of replicas to apply QAR.

#### Production dominates Consumption (*λ_p_ > λ_c_*)

In this case the producers just push the data to the closest replication node, whereas a consumption query first reaches the closest replica, which then routes the query to the rest of replication nodes (using the combing routing) that finally reply back with the requested information if any is available. Therefore, we can use the same model than in the previous case, but interchanging consumption and production costs we assume that the distance traveled by the query to all replicas is the same as the distance of all replies. We already mentioned when introducing ToW (see Section 5.3.) that this is only possible if aggregation is assumed. Therefore, in order to fairly compare our proposal with ToW we follow the same assumptions considered by ToW..

Then the consumption and production costs and $N_{r}^{*}$ are in this case:

$$\begin{array}{c} T_{c}\left(\lambda_{r}\right)=\alpha \lambda_{c}A\frac{\delta }{\sqrt{\lambda_{r}}}+\alpha \lambda_{c}A\left(\lambda_{r}A-1\right)\frac{1}{\lambda_{r}}\text{bits-m}/\text{sec},\\ T_{p}\left(\lambda_{r}\right)=\beta \lambda_{p}A\frac{\delta }{\sqrt{\lambda_{r}}}\text{bits-m}/\text{sec},\\ N_{r}^{*}=\lambda_{r}^{*}A=\delta \frac{\beta \lambda_{p}}{\alpha \lambda_{c}}-(1-\delta ) \end{array}$$

This model (*λ_p_* > *λ_c_*) is symmetric to the previous one (*λ_c_* > *λ_p_*) thus, it could be also validated by Figure 7.

### 7.2. Performance Evaluation

This section first compares the network cost in terms of hops when the most relevant multi-replication solutions, ToW and multi-replication GHT, are compared with QAR. To be fair we need to compare ToW write-one-query-all mode and QAR production-dominates-consumption mode to the multi-replication GHT functionality. We remember that in GHT with multiple replicas producers push the event data to the closest replica, then consumer queries need to reach all the replicas in order to retrieve the information. In addition, it must be highlighted that GHT with multiple replicas uses the hierarchical routing described in Section 5.1., whereas ToW and GHT use the combing routing introduced in Section 5.3..

**Figure 8.**
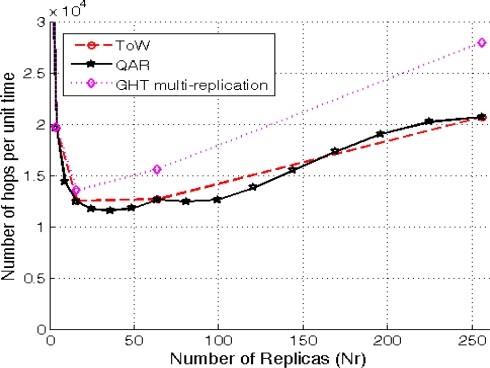
*N_c_* = 20, *N_p_* = 2000, *A* = 1,000 × 1000, *N* = 5,000, *Tx* = 60, Iterations = 50.

We simulate a sensornet with an area *A* = 1,000 × 1,000 square units, *N* = 5,000 sensors deployed at random and a transmission range *Tx* = 60 distance units. In order to simulate the traffic load among the 5,000 sensors, we randomly select 20 consumers and 2,000 producers. Consumers and producers generate traffic in a regular basis, that is, each consumer produces 1 query per unit time and each producer generates 1 data event per unit time. So that, we measure the traffic load in total number of hops in the sensornet per unit time using different number of replicas for each solution (*i.e.*, 1, 4, 16, 64 and 256 for ToW and GHT with multiple replicas, and 1, 4, 9, 16, 25, 36, 49, 64, 81, 100, 121, 144, 169, 196, 225 and 256 for QAR that, as it has already been described, has more granularity). The results shown in Figure 8 are the average traffic values (in number of hops per unit time) over 50 different scenarios for each number of replicas being evaluated.

The first conclusion is that GHT with multiple replicas is the worst solution. The hierarchical routing when using several replicas incurs in a higher cost than the combing routing, that is why in all the cases GHT with multiple replicas has the highest traffic cost. On the other hand, the figure shows a first approach to the granularity of QAR compared to the other solutions. QAR can choose up to 9 different number of replicas between 64 and 256, whereas ToW and GHT with multiple replicas only 2. Finally, it can be seen that in case of choosing the best number of replicas that minimizes the overall traffic (16 for ToW and 25 for QAR), QAR is the best solution.

After, demonstrating that QAR and ToW outperforms GHT with multiple replicas. We will analyze and compare the performance for different *λ_c_/λ_p_* and *λ_p_/λ_c_* ratios using the optimal number of replicas, $N_{r}^{*}$, provided by ToW and QAR analytical models. We compare the traffic load in number of hops for GHT with a single replica, ToW (with *k* = 1) and QAR in the same scenario. We cannot include GHT with multiple replicas in this evaluation because the authors do not propose any model or heuristic mechanism to calculate the optimal number of replicas to be used.

#### Consumption Dominates Production (*λ_c_* > *λ_p_*)

We evaluate a scenario with an area *A* = 1,000 × 1,000 square units, where *N* = 5,000 sensors have been deployed at random, and a transmission range *Tx* = 60 distance units. The ratio *λ_c_/λ_p_* is varied from 1 to 200. For each particular value we provide the average result obtained from 100 simulated scenarios. Finally, we consider the value of *α* to be twice of *β*, since each query needs a reply message, while production events just generate a single message. Under these conditions, we measure the extra overall network traffic (in number of hops) generated by ToW in front of QAR.

**Figure 9.**
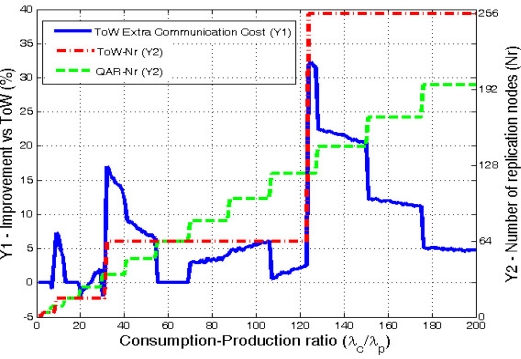
Extra communication cost produced by ToW in front of QAR when consumption dominates production in Y1 axis. Number of replicas used for each approach in Y2 axis (A = 1,000 ×1, 000, *N* = 5, 000, *Tx* = 60, *Iterations* = 100).

Figure 9 clearly shows that QAR is more adaptive than ToW because the number or replicas increases in smaller steps than ToW. Moreover, QAR is the one that minimizes the communication cost. ToW needs in average 7.56% more extra hops in the communications. If we look carefully at the results, they show that the improvement is specially significant when ToW model decides to increase the number of replicas. Then, during the cases where ToW uses *d* = 2 (16 replicas) the results show a maximum extra communication cost of 7.28% compared with QAR. In the cases where ToW chooses *d* = 3 (64 replicas), QAR improves ToW in average a 5%, with a maximum of a 17%. Finally, the case when ToW selects *d* = 4 (256 replicas) demonstrates that applying ToW with high ratios is not good enough due to its low adaptivity. In this case the maximum extra cost is 32.3% being the average gain of QAR a remarkable 13.5%.

It must be noted, that in some few cases ToW slightly outperforms QAR, but never goes further than a 2% improvement. This happens in very few cases where our model, which is a distance-based one, does not always provide the best number of replicas in terms of hops, but the one in distance that leads to a similar result in hops, but not always optimal.

As a side note, the original DCS proposal having a single replica has an average cost 3 times (300%) greater than our solution. This further demonstrates that multi-replication when applying Data Centric Storage is a must.

#### Production Dominates Consumption (*λ_p_ > λ_c_*)

We use a scenario with the same parameters than in the previous case. Again, the inverse ratio *λ_p_/λ_c_* changes from 1 to 200.

**Figure 10.**
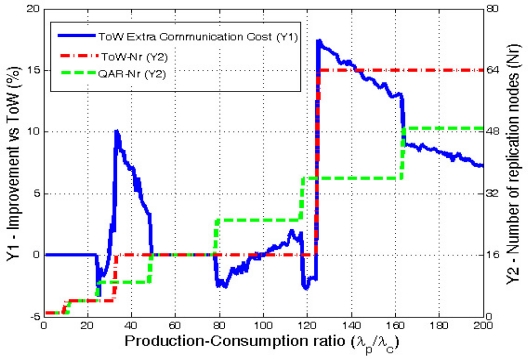
Extra communication cost produced by ToW in front of QAR when production dominates consumption in Y1 axis. Number of replicas used for each approach in Y2 axis (A = 1,000 ×1, 000, *N* = 5, 000, *Tx* = 60, *Iterations* = 100).

First of all, it must be noted that in this case increasing the number of replicas requires a higher *λ_p_/λ_c_* ratio than the *λ_c_/λ_p_* ratio required in the previous section. This is why in this case the ToW solution only goes up to 64 replicas (d = 3) while QAR reaches 49. Figure 10 shows the extra communication traffic that ToW generates when compared with QAR.

In this case the average extra cost generated by ToW is a 4.8%. Analyzing the results for the different values of *d* chosen by ToW, when *d* = 1 the improvement is not very significant. However, when ToW moves to *d* = 2, it produces a maximum extra cost of 10.15%. In the case of *d* = 3, QAR improves ToW in a 11.45% in average, reaching a maximum of 17.5%.

Again, there are some few cases when ToW outperforms QAR, but the difference is always lower than a 3%. The reason why this happens is the same as in the previous case.

When comparing our solution versus the original DCS with a single home node, the average extra communication cost produced by GHT is 145%.

We can conclude that the quadratic evolution of QAR is much more adaptive than previous solutions in the literature that follows a 4*^d^* grid structure replication like ToW or the original multi-replication GHT. QAR improves the average network overall communication traffic and reaches peak traffic reduction of 32% in front of ToW, which has been demonstrated to be less adaptive to network conditions. In addition, our solution does not add any additional complexity to the replication mechanisms proposed in the literature.

## 8. Conclusions and Research Challenges

This paper has presented the most relevant works in Data Centric Storage (DCS) for Wireless Sensor Networks (WSNs) including routing, multi-replication, storage policies, *etc.* Table 3 summarizes the main proposals and their contributions in the field of Data Centric Storage.

However, we believe that there are still some research challenges that need to be addressed.

A first research topic that still presents some challenges is the *storage analysis for DCS systems* (see Section 4.). Storage in DCS has been studied in very few works [27, 28] in the literature. These papers basically say that when the home node is saturated a new one is chosen, and later another one and so on. However, they do not consider that usually sensor data is only useful during a given time window. In addition, there could be many different event types in a sensornet, thus there are some probability that a given node is selected as home node (or replica) for several applications. Therefore, it would be very interesting to study the maximum number of applications (event types) that could be deployed at the same time in a WSN due to storage constrains.

In the area of multi-replication (Section 5.) we find different structured replication techniques, mainly represented by multi-replication GHT and ToW, but they are not adaptive enough due to its 4*^d^* replication scheme. In order to improve the adaptivity we have presented our QAR replication model, which increases the adaptivity by using a *d*^2^ replication scheme. In addition, QAR outperforms ToW and multi-replication GHT by reducing the overall network traffic (that has a direct impact on energy consumption and sensornet lifetime). However, all these works assume a square sensornet and are tied to a grid structure that requires some previous knowledge on grid number and size. We believe that an *unstructured replication scheme* that selects the optimal number of replicas without depending on a grid structure would reduce the complexity of the structured replication proposals, as well as improving its performance and adaptivity.

There is an inherent problem to DCS systems that has been only mentioned in [34] but not studied. This problem is the *unfair energy consumption distribution that DCS creates among the sensors* in static WSNs. It is clear that home nodes (in standard DCS scenarios) and replication nodes (in multi-replication DCS scenarios) incur in higher energy expenditures than other nodes within the network. Then, we believe that the role of the home node should change over the time in order to balance the energy consumption among all the nodes in the sensornet. We think that improving this energy balance will lead to extend the sensornet lifetime. Obviously, changing the replicas over the time balances the energy expenditures among the sensors, but it is not free of cost. Changing the replication nodes without information loss requires the data stored in the old replicas be moved to the new ones. Therefore, it should be studied what is the impact of this additional cost on the sensornet lifetime. In addition, a dynamic DCS system requires to define new mechanisms and protocols, and in order to be practical they should not add much complexity. It must be highlighted that this research challenge is only interesting in static WSNs, because if the sensors are mobile nodes, the home node (or replicas) changes over the time due to node mobility so no additional mechanism needs to be applied.

We have mentioned in this paper the autonomous Wireless Sensor and Actor Networks, where the network is self-operated without the necessity of a sink that connects it to the external world. Most of the research papers presented in this survey are focused on standard WSNs and assume that queries are generated from a single point, the sink. We believe that the interest on DCS will increase when autonomous WSANs become more relevant. In such a case, many different event types will be used in the network in different planes: control, management, data, etc. Therefore, DCS seems to be a very efficient storage solution for them.

Finally, DCS has been a very interesting topic for the research community and now it is time to start focusing in real implementations for DCS in order to demonstrate that it is a feasible system, able to support many applications running in parallel on top of it.

## Figures and Tables

**Table 1.** Cell selected in function of the event type and time slot.

	t = 0	t = 1	t = 5	t = 10	t = 15	t = 20	t = 30	t = 40	t = 50
*e*_0_	0	0	0	99	99	98	97	96	95
*e*_1_	1	1	1	0	0	99	98	97	96
*e*_2_	2	2	2	1	1	0	99	98	97
*e*_3_	3	3	3	2	2	1	0	99	98
*e*_4_	4	4	4	3	3	2	1	0	99
*e*_5_	5	5	5	4	4	3	2	1	0
*e*_6_	6	6	6	5	5	4	3	2	1
*e*_7_	7	7	7	6	6	5	4	3	2
*e*_8_	8	8	8	7	7	6	5	4	3
*e*_9_	9	9	9	8	8	7	6	5	4
*e*_10_	10	10	10	9	8	8	7	6	5

**Table 2.** Average producer and consumer costs in hop numbers for GHT, multi-replication GHT and Double Rulings.

	Producer Cost	Consumer Cost
GHT without replication	44.9	45.6
GHT with replication (*d* = 1)	172.4	17.4
Double Rulings	77.5	29.0

**Table 3.** DCS proposals summary.

**Proposals-Contributions**	**Routing**	**Multi-replication**	**Storage**
**GHT**	GPSR	single home node	-
**GHT with multiple replicas**	GPSR	Grid-based (4*^d^*)	-
**RendezVous Regions**	Region-oriented	single home node	-
**HVGR**	Landmarks + Region-oriented	single home node	-
**pathDCS**	Landmarks + Path-oriented	single home node	-
**Dynamic DCS**	GPSR	single home node	Change home node over the time
**Grid-Based Dynamic**	Geographic Routing + local decision	single home node	Change home node when saturated
**Resilient DCS**	GPSR	Grid-based z cells different type of queries	-
**ToW**	GPSR + Combing routing	Grid-based (4*^d^*) optimal d	-
**Double Rulings**	Circumference Routing	Circumference replication	-
**QAR**	GPSR + Combing routing	Grid-based (*d*^2^) optimal number of replicas	-
